# Emerging Zoonotic Infections, Social Processes and Their Measurement and Enhanced Surveillance to Improve Zoonotic Epidemic Responses: A “Big Events” Perspective

**DOI:** 10.3390/ijerph19020995

**Published:** 2022-01-17

**Authors:** Samuel R. Friedman, Ashly E. Jordan, David C. Perlman, Georgios K. Nikolopoulos, Pedro Mateu-Gelabert

**Affiliations:** 1 Department of Population Health, NYU Grossman School of Medicine, New York, NY 10016, USA; 2 Center for Drug Use and HIV/HCV Research, New York, NY 10003, USA; ejordanashly@gmail.com (A.E.J.); perlmandavidc@gmail.com (D.C.P.); pedro.mateu-gelabert@sph.cuny.edu (P.M.-G.); 3 Department of Infectious Diseases, Icahn School of Medicine, Mount Sinai Beth Israel, New York, NY 10003, USA; 4 Medical School, University of Cyprus, 1678 Nicosia, Cyprus; gknikolopoulos@gmail.com; 5 Graduate School of Public Health and Health Policy, City University of New York, New York, NY 10027, USA

**Keywords:** zoonoses, big events, surveillance, sociobehavioral surveys

## Abstract

Zoonotic epidemics and pandemics have become frequent. From HIV/AIDS through COVID-19, they demonstrate that pandemics are social processes as well as health occurrences. The roots of these pandemics lie in changes in the socioeconomic interface between humanity and non-human host species that facilitate interspecies transmission. The degree to which zoonoses spread has been increased by the greater speed and extent of modern transportation and trade. Pre-existing sociopolitical and economic structures and conflicts in societies also affect pathogen propagation. As an epidemic develops, it can itself become a social and political factor, and change and interact with pre-existing sociobehavioral norms and institutional structures. This paper uses a “Big Events” approach to frame these processes. Based on this framework, we discuss how social readiness surveys implemented both before and during an outbreak might help public health predict how overall systems might react to an epidemic and/or to disease control measures, and thus might inform interventions to mitigate potential adverse outcomes or possibly preventing outbreaks from developing into epidemics. We conclude by considering what “pathways measures”, in addition to those we and others have already developed, might usefully be developed and validated to assist outbreak and epidemic disease responses.

## 1. Introduction

Zoonotic pandemics and emerging infectious disease highlight the challenges that emerge from varying interfaces and interactions among humans and non-human organisms in environments. Some of the changes in interaction patterns are caused by environmental changes due to global climate change which alters the environments in which human and other organism interact [1,2,3,4]. Other gradual or sudden changes in human social processes, such as changes in the meat industry or military conquests can also lead to zoonotic pandemics and novel human infectious diseases if they lead to novel encounters with non-human animals and other organisms [5,6,7,8,9,10,11,12,13]. Theoretic frameworks for the study of zoonotic infections that incorporate measures of human social processes can help us understand, predict and address zoonotic pandemics.

We have previously developed a “Big Events” framework for research and interventions on pandemics, including zoonotic pandemics, that create, or occur under, situations of social emergency and interact with pre-existing social structures and processes [14,15]. We developed this perspective out of analysis of HIV/AIDS outbreaks that took place in the countries of the former Soviet Union after the dissolution of the USSR; in South Africa after the end of apartheid; and in Indonesia after the economic crisis and overthrow of the dictatorship in the late 1990s [7,15,16,17,18,19,20,21,22,23,24,25,26,27,28,29,30,31,32,33,34]. The global HIV epidemic is a zoonotic pandemic that arose from the transmission of a primate retrovirus to humans. This transmission took place due to new patterns of human-primate interaction during the European conquest and exploitation of Africa, and HIV then spread from colonial Africa across the world during the mid-20th century and beyond [35,36,37]. We refined this framework based on what we learned by applying it to an HIV upsurge which occurred in Athens, Greece, amidst financial and economic crisis [15,23]. Importantly, such HIV outbreaks appear to be historically and contextually *contingent*–similar events in Argentina early this century did not lead to an HIV outbreak despite there being a sizable number of infected people who could have served as seeds for a wide epidemic [14,15].

As the COVID-19 pandemic has made clear, zoonotic pandemics can create economic and social crises as well as a generalized sociopolitical discontinuity in society as in South Africa during the late 1990s and for some years thereafter [12,38]. Historically, in the mid-400s BC a zoonotic epidemic (which may been either plague or typhus) devastated the Athenian city-state causing major social and economic disruption and probably contributing to its collapse and loss in the Peloponnesian war, and in the 6th Century AD, plague epidemics (caused by *Yersinia pestis*) caused sociopolitical crises that contributed (along with other factors) to the decline of the Roman Empire [39]. Subsequent plague epidemics in the 13th and 14th centuries, introduced by increased trade among geographic regions, caused massive mortality, demographic and social upheavals that contributed to the decline of feudalism in Europe and to the rise of capitalism as the dominant mode of social production in Europe (and subsequently elsewhere) [38,40,41,42,43]. The invasion of the Americas by European settler-colonialists and slave traders in the 16th century, and critically the zoonotic pathogens which traveled with them, led to massive disruption and destruction of indigenous cultures and political systems [38,41]. A cholera epidemic which occurred in Paris in 1832 interacted with pre-existing social tensions to contribute to the Paris Uprising of that year and to subsequent government repression [44]. The 1918 influenza pandemic occurred in the context of the human morbidity mortality and destruction of built environments caused by World War I, and caused further massive social disruption in Europe, Russia, the US and elsewhere [45,46]. A recent International Monetary Fund working paper found a strong, but complex, relationship between epidemics and ‘social unrest’ events in the period 1990–2019 [44]. These selected examples demonstrate the long history of pandemics both arising in the context of sociopolitical stresses, and causing significant sociopolitical disruption.

Standard approaches to understand the epidemiology of pandemics and to inform planning and mitigation strategies are often based on forms of the agent-host-environment model [47]. The agent-host-environment model used in these standard approaches typically incorporates measures of individual human biologic and behavioral characteristics, group-level human characteristics (e.g., population prevalence of pathogen, or ‘herd’ immunity), measures of pathogen (agent) characteristics (e.g., virulence and transmissibility) and measures of selected environmental (contextual) characteristics. While these factors are necessary to understand epidemic curves (i.e., trajectories of epidemics over time), they do not fully account for how the agent, the host or the environment *interact* and change over time. Neither the standard measures nor the agent-host-environment model in-and-of-itself adequately reflects or articulates many important social processes which are both socially plausible (where social plausibility is a form of face validity analogous to biologic plausibility) and available data suggest to be key factors that shape how pandemics change over time [48] A framework to understand zoonotic epidemics should more fully consider the time-varying and dialectical interactions among pathogenic evolution, medical interventions, policies, social and economic processes and conditions, and human behavior. The “Big Events” framework, which is the focus of this paper, could be useful in understanding and seeking to prevent and mitigate such epidemics.

One key element of this framework is that it includes many social processes and relationships that are rarely considered in epidemiologic or prevention research and policy. These include processes and structures that can currently be measured, as well as others for which measurements could be developed. Measuring these processes and how they change before and during an epidemic could enhance the understanding of pandemic trajectories, as well as their prediction and mitigation. We propose that a number of novel measures should be included in a system of “social readiness surveys” that ideally should be begun and maintained over time before, during and between epidemics.

We developed a “pathways model” based on our and others’ previous work to explain how Big Events affect social, behavioral and economic processes that in turn affect HIV incidence rates or other epidemiologic outcomes [27,29,30,31,32,33]. These pathways include a large number of social processes for which no then-existing measures existed. We conducted mixed methods research to develop and validate a number of survey scales and free-standing items to measure social processes including intergenerational normative disjuncture [30], increases in the magnitude or sources of dignity attacks on various groups in the population [31], of individual and group altruism and solidarity [32], and other important social process constructs [29,30,49].

As a considerable body of past research shows [10,13,50], there is every reason to predict that the combined and interacting impacts of economically-driven human incursions into, and the redevelopment of, forestlands, savannahs, and other areas where potential vectors and potential emerging pathogens exist; the ecological interface between industrialized animal husbandry and naturally occurring wildlife; the commodification and privatization of sources of water and of aquatic food chains and destruction of aquatic ecosystems; and global climate change will lead to major future zoonotic pandemics that may well be socially disruptive [51,52,53]. Further, as has occurred in the United States, France, and Brazil during the COVID-19 pandemic, pandemic related socioeconomic disruptions may interact with pre-existing social and political inequities and/or pre-existing political divisions to render public health policy and practice a contentious political issue. This then may feed back to weaken public health policies and contribute to disease spread. In addition, the COVID-19 pandemic and responses to it have led to delays in elections, some of which have been contentious and contested (e.g., in Ethiopia) and have been used a means of justifying repression and suspension of the rule of law (e.g., in Uganda in which the party in power held large rally events but cited the pandemic as a putative rationale to prevent opposition party rallies) [54].

In this brief article, we will present a preliminary framework and model of how these processes might form an interactive system in a nation or other jurisdiction. Based on this model, we will discuss how social readiness surveys implemented both before and during an outbreak might help public health actors predict how the overall system might react to an epidemic and/or to disease control measures and thus might help inform interventions to mitigate potential adverse outcomes (including possibly preventing outbreaks from developing into epidemics). We will conclude by considering what additional “pathways measures”, in addition to those we and others have developed, might usefully be developed and validated by researchers to be incorporated in outbreak and epidemic disease responses.

## 2. A General Framework of Response to a Zoonotic Outbreak

**Figure 1:**
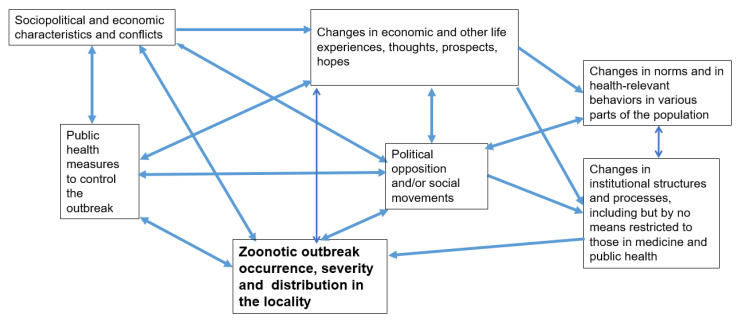
General model of zoonotic outbreaks in a locality in a Big Events framework and what pre-existing social surveillance might be useful. Social surveillance should consider measuring all of the social variables referred to in the Figure. It should be noted that more than one Big Event can take place concurrently. If this occurs, complex interactions may lead to greater peril, depending on the social readiness to meet these other Big Events.

Experience with both HIV/AIDS and COVID-19 makes it clear that the effects of a zoonotic disease outbreak on a population depend on pre-existing sociopolitical conditions (a term that includes economics as well), public health responses, the ways in which the outbreak and responses to it affect the life experiences, thoughts and hopes of different sections of the population, and changes in the norms and health-relevant behaviors of various parts of the population [27]. (See Figure 1) Importantly, all of these considerations can precipitate mass political and social movements, shaped to varying degrees by pre-existing patterns of political conflict, stigmatization, household organization, economic inequality and exploitation, and institutionalized oppression or subordination [27,55,56]. In the case of COVID-19 in the United States (US), for example, the pandemic has caused far greater mortality for Native American, Latinx, and Black people than for white people; these disparities were also reflected both in politics (where the great majority of anti-vaccination and anti-masking activists have been white people, many of them overt racists) and perhaps in the huge antiracist demonstrations across the US in the summer of 2020 [14,57,58].

COVID-19 in the US (and elsewhere) has led to more financial hardship and job loss among women than men (particularly comparing those with children with those without or those who are immigrant with those who are not), with Latinx, Black, and indigenous females further impacted than their White female counterparts [59,60,61,62]. It is not clear what the political and long-range impacts of these disparities will be, nor of how they may influence change in the pandemic.

Social-level responses during times of zoonotic disease outbreaks can affect institutional structures and processes, including the funding, personnel, and structure of health departments, other health care and prevention services, other social and economic support systems, and police and the policies implemented to address the zoonotic outbreak, which can affect the effectiveness of responses and can sometimes lead to inequities in how groups experience outbreak or pandemic conditions [63,64,65]. Further, there is a considerable degree of interaction and reverse causation in these causal chains.

The Big Events framework is complex. Other global and local crises—specifically including global climate change and its local manifestations and the possibility of more than one, sometimes interacting, zoonotic outbreak at the same time and place—can shape these complex processes and potentially affect the pattern, distribution and severity of human and non-human zoonotic disease.

The current Big Events framework—and the social readiness surveys based on it—may be limited by their genesis in studying the HIV/AIDS and (to an extent) the COVID-19 pandemics. Future modifications should increase their power to explain other potential zoonotic pandemics such as those driven by vector-borne or waterborne disease transmission. Further specification of the economic processes involved in pandemics, such as supply-chain disruptions, should be based on understanding economic processes (which are best understood as social relations that are continually changing.) Production, finance and trade are all forms of social relations that can greatly shape emerging epidemics and efforts to mitigate, control or eliminate them [38,66].

Zoonotic pandemics have long been linked to trade routes including infections such as the plague, small pox, and HIV [38,66]. Commercial transportation and leisure travel (e.g., via cruise ships) were important venues for COVID-19 and SARS-1 disease transmission. Similarly, the congruence of human, birds and pigs in locations used by humans for commercial food production, such as rice paddies, brings these animal populations into contact and creates niches in which influenza variants emerge [1,62]. Food production in meatpacking plants also provided venues for widespread COVID-19 transmission, a process that was, in the USA at least, closely linked with racist social and economic practices that concentrated subordinated race/ethnicities in meatpacking jobs [67,68].

One measure of the degree to which governments have adopted neoliberal trade, taxation and other policies is the “economic freedom index” developed by the conservative Heritage Society. Although this measure has not, insofar as we are aware, been used to study zoonotic epidemics, it did prove useful in a study of the “non-communicable epidemic” of obesity. Higher “economic freedom” increases or ‘easier’ trade was associated with the prevalence of obesity, plausibly though increased trade in and increased consumption of fatty foods. This and analogous measures of socially mediated trade relationships could be explored for their relationship to zoonotic pandemics. Similarly, one could imagine measures of trade that relate volume on specific trade routes to global patterns of pathogen dissemination [69,70].

As an additional complication, pathogens constantly mutate at the genetic level due to errors in the replication process. These mutations may accumulate and generate strain variations which may be intrinsically more or less transmissible, more or less virulent, and more or less able to escape existing individual- and population-level immunity. These new strain variations are then subject to selection pressures in specific niches which may favor their replication and spread in that niche. Interventions such as antiretroviral treatment (e.g., for HIV/AIDS) or vaccination of human populations (for various infections) exert selection pressure on both original and newly emerged strains and may result in the selection and dissemination of new, and possibly more virulent or more transmissible strains. Further, as Paul Ewald and others have discussed, social structures and changing social contexts can create “social niches” that favor the rapid emergence and dissemination of pathogen variants [71,72,73,74]. As an example, Ewald discussed how the niches created by the social organization of military trenches and military medicine in World War I may have contributed to the emergence and dissemination of more virulent influenza strains. For HIV, such social niches probably included multi-person psychoactive drug injection sites, some brothels or sex worker “strolls”, and the multi-person use of syringes in clinical settings in some parts of Africa in the 1950s and after, and sex parties and other multi-partner settings prominent in gay clone cultures that developed in the US in the 1970s [71,73,75,76,77].

## 3. Example: The Covid-19 Outbreak in the US in a Big Events Framework

In order to illustrate in further detail how the general framework in Figure 1 might be applied, we discuss how upstream domains (boxes) highlighted in the framework have shaped the development of the Covid-19 epidemic in the US.

*Pre-existing sociopolitical and economic characteristics and conflicts* led to political polarization within multiple domains which directly relate to the COVID-19 epidemic and such responses to it as assessments of infection importance [78], “stay-at-home” measures [79], food production [80], regulation of religious gatherings [81], safety and effectiveness of medical treatments for COVID-19 infection [49,82], and “mandates” of both mask wearing [83] and COVID-19 vaccination [84]. These conflicts were reflected in the media, contributing as much to confusion and misinformation as to accurate health literacy (although differentially so for different subpopulations) [85,86]. This confusion interfered with the implementation of consistent and coordinated responses to the epidemic. These dynamics further contributed to a widely inequitable and unequal implementation of *public health and public policy responses* across US states [87] resulting in inconsistent and contradictory public health messages and in states, agencies and institutions competing for Federal support, as for example, in access to personal protective equipment, respirators, and medications and vaccines made available through emergency use authorizations prior to full product approval [88]. This early politicization emboldened political opposition to many public health responses. Social movements such as white nationalism and sections of anti-vaccine movements expanded their reach aided by the use social media platforms by humans and by the use of social media bots and artificial intelligence on these platforms [78,85]. At times anti-vaccine reactions manifested in physical threats to public health officials [89] and sometimes in armed demonstrations and violent attacks in state capitols [90,91].

This on-going multi-pronged polarization led to *various patterns and changes in social norms and health relevant behaviors.* Gardarian, et al. (2021) found that political party affiliation consistently predicted differences in health behaviors, beliefs, and attitudes related to COVID-19 [92]. Similarly, COVID-19 vaccination uptake has been consistently higher in counties that voted Democratic in the US presidential election compared with those which voted Republican, with the gap widening over time [93]; COVID-19 case rates, morbidity and mortality were also distributed differentially similarly.

Other changes caused by interactions across these domains were less contentious. These included some changes in behavioral practices such as handshaking and changes in institutional structures with possibly lasting impact such as normalization of remote working for occupations relying on primarily mental rather than manual labor (a COVID-19 response that may have exacerbated inequities in COVID-19 risk based on occupation and linked to race, ethnicity, gender and class inequities in employment and occupation), [94] expansion and acceptance of telemedicine [95,96], and reductions in the authority of many local and state health departments, including the dissolution of at least one health department (Tri-County, in Colorado) and rancorous policy disputes and elections focused on masks in schools.

The HIV/AIDS epidemic, thanks in great measure to self-mobilization of the most affected marginalized groups [97], led to medical advances such as redefining AIDS to include HIV-related conditions that affect women and people who use drugs, expanded access to clinical trials and accelerated approval of effective medicines. Activists’ efforts also led to public health measures such as promotion and distribution of condoms, harm reduction services and greater mainstream acceptance of LGBTQ communities and extension of their rights [98,99]. In contrast, the COVID-19 pandemic might have arguably weakened the ability of the US to respond to future epidemics by undermining its collective ability to enact and accept public health laws [100] that might be perceived (and politically promoted) as an attack on individual liberties.

## 4. Social Readiness Monitoring and Other Forms of Preparation and Learning

Very little research has been conducted on how the various factors in our pandemic framework interact with each other as a dynamic system. One reason is that many of the constructs involved have rarely been measured. For some of them, validated measures may not exist. Our team developed a set of “pathways measures” to describe changing local social conditions and including emerging new norms that might shape how Big Events or macrosocial “structural” interventions would affect HIV/AIDS epidemics [27,30,31,32,33]. For example, we used one set of these measures (i.e., of norms that encouraged risky behaviors including use of unsterile injection equipment) in Athens, Greece following an outbreak of HIV among people who inject drugs (PWID) between 2011–2013 [101,102]. Although numbers of diagnosed HIV infections had decreased when we collected these data (2013–2015), both overall socioeconomic conditions and the norms of many people who inject drugs favored high risk drug injection contexts and behaviors. Unsurprisingly, in 2018–2020, the prevalence of HIV among PWID in Athens had increased to 22% from 14% in 2013, and there is also evidence for high transmission in Greece’s second largest city (Thessaloniki) [103].

Further, social movement scholars and others have developed methods to study political and social movements, and both large population level surveys (e.g., census data, national labor statistic surveys) and market research often measure other variables highlighted in Figure 1. Insofar as we are aware, little-to-no work has yet combined these sets of variables with data on pathogen phylogenetics, the emergence, diversity and dissemination of pathogen variants and epidemic curves, and relevant outcomes in human (or non-human) populations. This is an area of potentially important future work with direct implications to both public health and public policy.

As discussed above, we suggest that it might be very useful to establish integrated real-time social readiness monitoring and surveillance systems for as many of these variables as can be measured to allow development of both predictive and explanatory models to help prepare responses for potential outbreaks and to prevent or respond quickly to potential outbreaks. Social readiness surveys could be combined with other population-level census and economic census data, along with pathogen surveillance systems, to provide measures of many of the variables in our framework. If such systems can be established in enough localities, it would also enable researchers to begin to understand the interactions of the complex system depicted in Figure 1. The relevance of different “pathways variables” may vary depending on characteristics of the pathogen in question. Some of the measures we developed to study HIV epidemics may be useful for other zoonotic infection epidemics as well [29,30,32]. For respiratory infections such as COVID-19, patterns of close proximity at workplaces (particularly indoor, poorly ventilated workplaces), housing (particularly crowded, poorly ventilated housing, including congregate settings such as shelters, homes for aged populations, prisons and jails), as well as the norms, institutions and laws that affect these, will be crucial measures [55,104,105,106,107,108]. For vector-borne infections or water-borne infections, still other pathways variables might need to be developed. We expect that other pathways variables, such as those on altruism and patterns of solidarity and conflict within a population, are relevant to all pandemics.

In conclusion, we suggest that establishing both pathogenic real-time surveillance systems and social readiness surveys could help the human response to zoonotic outbreaks by more fully considering changes in human social process that affect, respond to, and form part of the environment in which human zoonotic pathogens emerge and disseminate. The transdisciplinary nature of the Big Events framework and of forms of social readiness monitoring bring challenges. Yet, the development of measures and methods of enhanced social readiness monitoring, and pandemic surveillance models to inform public health and societal responses, hold promise for improving human responses to current and future pandemics.
